# A Comparative Study of the Anti-Obesity Effects of Dietary Sea Cucumber Saponins and Energy Restriction in Response to Weight Loss and Weight Regain in Mice

**DOI:** 10.3390/md20100629

**Published:** 2022-10-01

**Authors:** Lu Wen, Rong Li, Ying-Cai Zhao, Jin-Yue Yang, Xiao-Yue Li, Chang-Hu Xue, Tian-Tian Zhang, Yu-Ming Wang

**Affiliations:** 1 College of Food Science and Engineering, Ocean University of China, Qingdao 266003, China; 2 Laboratory for Marine Drugs and Bioproducts, Pilot National Laboratory for Marine Science and Technology, Qingdao 266237, China

**Keywords:** energy restriction, sea cucumber saponins, weight loss, weight regain, insulin resistance, inflammation

## Abstract

Dietary supplementation of sea cucumber saponins and calorie restriction have been proved to be effective in alleviating obesity, but the differences of anti-obesity effects between sea cucumber saponins and energy restriction during weight loss and weight regain are still unknown. In the present study, high-fat-induced obesity mice were randomly divided into three groups, including a high-fat diet group (HF), an energy restriction by 40% group (HF-L), and a sea cucumber saponins group (HF-S), to compare the effects of dietary sea cucumber saponins and energy restriction on the weight, glucose, and lipid metabolism of obese mice during weight loss and weight regain. The results showed that dietary 0.06% sea cucumber saponins and limiting energy intake by 40% had the same weight loss effect. Interestingly, sea cucumber saponins could alleviate impaired glucose tolerance and insulin resistance caused by obesity. In addition, the inhibited SREBP-1c mediated lipogenesis might lead to the alleviation of weight regain after resuming the high-fat diet even when sea cucumber saponins were no longer supplemented. In contrast, limiting energy intake tended to promote lipid synthesis in the liver and white adipose tissue after restoring a high-fat diet, and inflammation was also induced. The findings indicated that sea cucumber saponins could replace calorie restriction to prevent obesity and might be used as a functional food or drug to resist obesity and related diseases caused by obesity.

## 1. Introduction

Obesity is usually considered as a systemic disease caused by bad lifestyle and eating habits, which can cause damage to important functions of the human organism. Obesity is a risk factor for various diseases and is an independent cause of death [1]. The threat of obesity to public health lies in the prevalence of complications in obese people, such as cardiovascular disease, diabetes, osteoarthritis, and various cancers [2]. The increasing prevalence of overweight and obesity worldwide has exacerbated concerns regarding the health risks associated with this growing problem.

**Figure 1:**
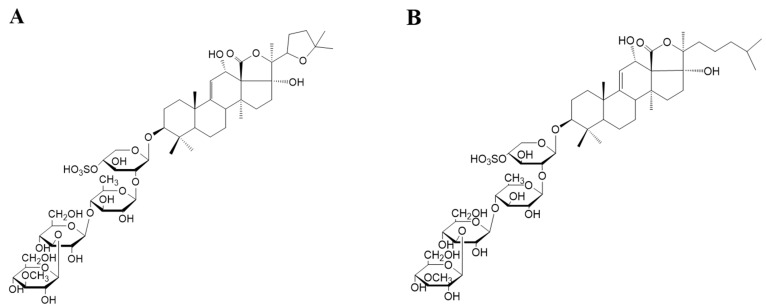
Structures of sea cucumber saponins: Holothurin A (**A**) and Echinoside A (**B**).

There is a strong rationale for the promotion of energy restriction and exercise to reduce weight in overweight and obese patients [3]. As is well known, exercise fits into the overall public health interests of low cost and readily available therapies for obese patients. However, obese patients are faced with practical difficulties such as insufficient exercise time, low public participation, and difficulty in adhering to exercise for a long period [4,5]. Moreover, there are various studies that have reported that it is harmful to return to a high-fat diet after energy restriction, although limiting energy intake does have a significant role in reducing body weight. When the forcible control of energy intake is lifted, it will lead to an abnormally high rate of weight regain [6]. Other studies have indicated that chronic energy restriction will lead to weight loss. However, it also increases the mRNA expression of orexigenic neuropeptides such as neuropeptide Y (NPY) and agouti-related protein (AgRP), which results in increased food intake and severe weight regain after stopping energy restriction [7]. Sea cucumber, belonging to the family of Echinodermata, is a type of precious marine food and an important medicinal resource in Asia. Sea cucumber saponins are the secondary metabolites of sea cucumber, and are glycosides of triterpene aglycones or steroidal aglycones [8]. Figure 1 shows the two main sea cucumber saponins Holothurin A (HA) and Echinoside A (EA) [9]. Sea cucumber saponin, (SCS) as a natural active ingredient, has been confirmed to have lipid-lowering properties. Dietary supplementation with sea cucumber saponins has been seen to reduce hepatic triglyceride and cholesterol concentration in rats with orotic acid-induced nonalcoholic fatty liver disease (NAFLD) [10]. Notably, it has been reported that dietary administration of sea cucumber saponins significantly inhibited lipid synthesis and accelerated fatty acid β-oxidation and glycolytic pathways by affecting the SIRT1 signaling pathway and its downstream genes, thereby reducing the weight gain, liver, and serum lipid levels of mice fed with a high-fat diet [11]. However, the effect of sea cucumber saponins on weight regain in mice after resuming a high-fat diet is unknown. There is also no information regarding the differences of effects between sea cucumber saponins and energy restriction during weight loss and weight regain.

In this study, a high-fat-induced obesity mice model was established to compare the effects of dietary supplementation of sea cucumber saponins and energy restriction on the weight, glucose, and lipid metabolism of obese mice during weight loss and weight regain. What is more, the possible mechanism of lipolysis and lipid synthesis was expounded.

## 2. Results and Discussion

### 2.1. Effects on Food Intake, Body Weight, and Visceral Weight Ratio

There was little difference in food intake between the HF-S group and the HF group during weight loss and weight regain, indicating that sea cucumber saponins did not cause changes in the appetite of mice (Table 1). After two weeks of dietary supplementation with sea cucumber saponins and energy restriction, the weight changes of the HF-S group and the HF-L group were significantly lower than those of the HF group by 1.85g and 6.38g, respectively. Interestingly, when all three groups resumed the high-fat diet within one week during weight regain, the weight of the mice in the HF-L group rebounded by 19.5%, and the HF-S group only rebounded by 7.1%. Although the weight loss effect of dietary 0.06% sea cucumber saponins was not as good as limiting 40% calorie intake, when the forcible control of energy intake was relieved, sea cucumber saponins significantly resulted in a lower rate of weight regain than energy restriction. Figure S1 shows the weight change during weight loss and weight regain. Moreover, there was no significant difference in the visceral weight ratio of liver, heart, kidney, and spleen among the three groups during the two periods of weight loss and weight regain (Table 1), indicating that neither energy restriction nor sea cucumber saponins supplementation caused damage to organs.

### 2.2. Effects on Adipose Mass, Morphology, and Mrna Expression of Fsp27 and PLIN1

**Figure 2:**
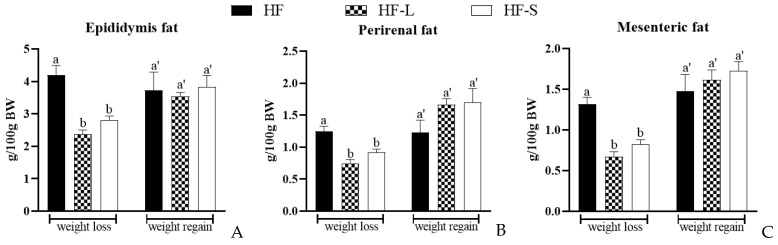

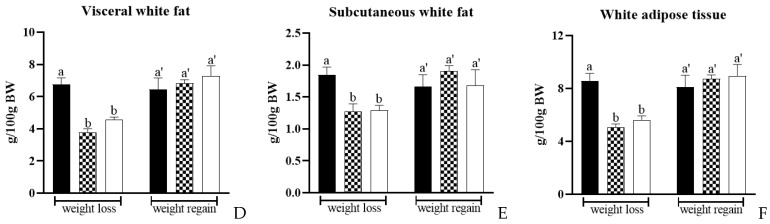
Effects of dieting and saponins on the weight of epididymis fat (**A**), perirenal fat (**B**), mesenteric fat (**C**), visceral white fat (**D**), subcutaneous white fat (**E**), and white adipose tissue (**F**) during weight loss and weight regain. Data reflect the mean ± SEM. Different letters a, b during weight loss and a’, b’ during weight regain indicate significant differences at *p* < 0.05. HF, high-fat group; HF-L, limited high-fat diet; HF-S, high-fat diet with saponins.

Compared with the HF group, both energy restriction and sea cucumber saponins significantly reduced the weight of epididymis fat, perirenal fat, mesenteric fat, visceral white fat, subcutaneous white fat, and white adipose tissue (Figure 2). Notably, there was no significant difference between the sea cucumber saponins and energy restriction groups in terms of reducing the weight of white adipose tissue during weight loss. One week after resuming the high-fat diet, the amount of white adipose tissue increased by 73% in the HF-L group and 59% in the HF-S group.

**Figure 3:**
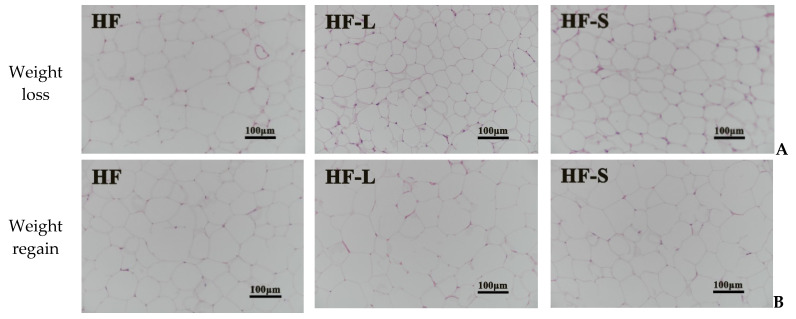

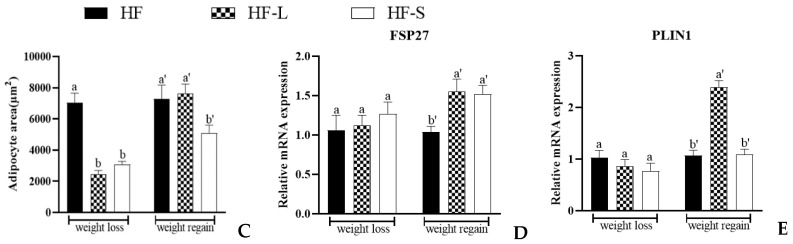
Morphological changes of the epididymal adipose tissue by H&E staining during weight loss (**A**) and weight regain (**B**). Epididymal adipocyte area (**C**). The mRNA expression of Fsp27 (**D**) and PLIN1 (**E**) in the epididymal adipose. Data reflect the mean ± SEM. Different letters a, b during weight loss and a’, b’ during weight regain indicate significant differences at *p* < 0.05. HF, high-fat group; HF-L, limited high-fat diet; HF-S, high-fat diet with saponins.

H&E staining reflected the morphological changes of the epididymal adipose tissue during weight loss (Figure 3A) and weight regain (Figure 3B). Figure 3C shows the epididymal adipocyte area. During weight loss, the size of adipocytes in epididymal white fat in the HF-L and HF-S groups was significantly smaller than that in the HF group. What is more, the size of adipocytes in the HF-L group was slightly smaller than that in the HF-S group. Significantly, it should be noted that one week after the restoration of high-fat diet, adipocytes in the HF-L group proliferated rapidly, and the size was close to that in the HF group and significantly larger than that in the HF-S group. It was reported that, when returning to the high-fat diet for two weeks, the size of adipocytes was larger than those fed the high-fat diet [12]. Our results showed that the adipocyte size of HF-L mice was similar to that of mice fed a high-fat diet one week after resuming the high-fat diet, which may be due to the fact that our weight recovery time was one week less than reported. The above H&E staining results show that both sea cucumber saponins and energy restriction can not only inhibit the increase of white adipose mass induced by a high-fat diet, but can also inhibit the hypertrophy of adipocytes. One week after the end of energy restriction, the size of adipocytes in the HF-L group increased rapidly compared with the HF-S group. Sea cucumber saponins may be used as natural food functional components to replace energy restriction and achieve weight loss.

Fat-specific protein 27 (Fsp27) localizes to the surface of lipid droplets, promotes lipid droplet fusion, and makes lipid droplets larger [13]. Perilipin 1 (PLIN1) promotes adipocyte proliferation and differentiation. Both Fsp27 and PLIN1 are related to adipocyte morphology. We investigated the mRNA expression of Fsp27 and PLIN1 in epididymal adipose tissue. Results showed that there was no significant difference in the mRNA expressions of Fsp27 and PLIN1 among the three groups during weight loss (Figure 3D,E). The mRNA expression of Fsp27 in the epididymal adipose tissue of the HF-L and HF-S groups were significantly higher than that in the HF group after resumption of the high-fat diet, and there was no significant difference between the two groups. At the same time, the visceral fat mass also increased in both groups. It has been reported that the Fsp27 protein level in the calorie-restricted group was 1.29 times higher than in the unrestricted group after a four-week calorie-restricted diet followed by a four-week normal diet [14]. Interestingly, the mRNA expression of PLIN1 in the HF-L group increased sharply during weight regain and was significantly higher than that in the HF group, while there was no significant difference between the HF-S group and the HF group. The PLIN1 protein is located in the outer layer of lipid droplets and prevents lipases in the body, such as hormone-sensitive lipase (HSL) and adipose triglyceride lipase (ATGL), from entering the lipid droplets [15]. These two enzymes are the main rate-limiting enzymes for lipolysis. Overexpression of PLIN1 results in lipid droplet fusion and triglyceride accumulation [16]. This may be the reason why adipocytes in the HF-L group grew faster than those in the HF-S group, from weight loss period to weight regain period.

### 2.3. Effects on the Expression of Epididymal White Adipose Tissue Genes Involved in Lipid Metabolism

**Figure 4:**
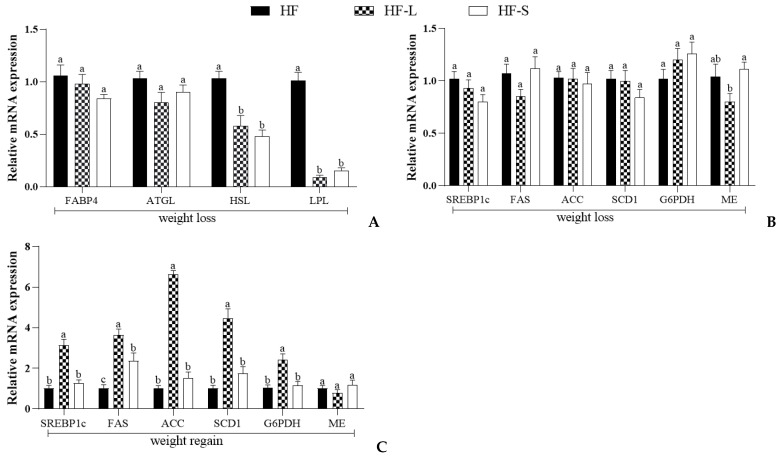
Effects of dieting and saponins on the mRNA expression of genes related to lipolysis and lipogenesis in the epididymal adipose tissue of mice. The mRNA expression of FABP4, ATGL, HSL, and LPL during weight loss (**A**). The mRNA expression of SREBP1c, FAS, ACC, SCD1, G6PDH, and ME during weight loss (**B**) and weight regain (**C**). Data reflect the mean ± SEM. Different letters indicate significant differences at *p* < 0.05. HF, high-fat group; HF-L, limited high-fat diet; HF-S, high-fat diet with saponins.

In addition to studying the expression of lipid droplet peripherin-related genes, we also explored the expression of epididymal lipolysis-related genes (Figure 4A) and lipid synthesis-related genes (Figure 4B) during weight loss, and the expression of epididymal lipid synthesis-related genes (Figure 4C) during weight regain. Fatty acid binding protein 4 (FABP4) is an adipokine that coordinates lipid transport in mature adipocyte, and its inhibition in obesity models shows weight loss and normalized insulin response [17]. Lipoprotein lipase (LPL) belongs to the lipase superfamily, and its main function is to provide tissue-utilized non-esterified fatty acids (NEFA) by breaking down triglycerides (TGs) in lipoprotein particles, such as chylomicrons (CM), very low-density lipoproteins (VLDL), and 2-monoacylglycerol, to provide energy [18]. ATGL and HSL are enzymes that play a role in lipolyzing and catabolizing stored TGs in lipid droplets [19]. ATGL hydrolyzes TGs into diglycerides (DGs), and HSL is a key neutral lipase that mainly decomposes DGs to monoglycerides (MGs) and free fatty acid (FFA) [20]. The results showed that there was no significant difference in the expression levels of fatty acid binding protein 4 (FABP4) and ATGL among the three groups during weight loss (Figure 4A). Notably, the mRNA expression of HSL in the HF-L and HF-S groups was significantly lower than that in the HF group by 43.7% and 72.8%, respectively. In addition, compared with the HF group, the expression of LPL in the two groups was decreased by 91% and 85.1%, respectively. In a population experiment, it has been reported that the caloric restriction was followed by a decrease in basal LPL activity to one fifth of the value recorded during the isocaloric diet [21]. The relative expression of HSL and LPL was not significantly different between the HF-L and HF-S groups. It has been reported that dietary ginsenoside-rich fermented ginseng berries for 16 weeks significantly inhibited the mRNA expression of HSL in white adipose tissue compared with that in the high-fat diet group [22]. In our previous study, it was found that there was no significant difference in the expression levels of HSL and LPL between the 0.06% sea cucumber saponin supplement group and the HF group after male KM mice were fed a high-fat diet for 3 weeks [23]. The difference in results might be attributed to the different experimental designs. Specifically, in our previous study, we mainly focused on the role of sea cucumber saponins in preventing obesity under a high-fat diet, and the simultaneous intervention of high-fat diet and sea cucumber saponins was conducted; in the present study, the focus was on the effect of sea cucumber saponins on weight loss, and the dietary high-fat diet led to obesity before the intervention of sea cucumber saponins.

Sterol regulatory element-binding protein (SREBP-1c) is an important transcription factor regulating de novo lipid synthesis, and its downstream key target response genes also include acetyl-CoA carboxylase (ACC), fatty acid synthase (FAS), malic enzyme (ME), and glucose-6-phosphate dehydrogenase (G6PDH) [10]. During weight loss, the relative expressions of SREBP1c and its directly regulated downstream target genes FAS and ACC, and its indirectly regulated downstream target gene G6PDH, were not significantly different among the three groups (Figure 4B). The expression of stearoyl-CoA desaturase 1 (SCD1) was positively correlated with the desaturation index, and there was no difference in its expression among the three groups. ME is accompanied by the reduction reaction of NAD(P)+ in the process of regulating malate metabolism [24]. When G6PDH plays a role in regulating the pentose phosphate pathway, its reducing equivalent is also stored in the form of NADPH [25]. The NADPH produced in the two forms is the extension of the fatty acid chain, an important source of the reducing power required. Notably, compared with the HF group, there was no significant difference between the expression level of ME in either the HF-L and the HF-S groups. However, the expression level of ME in the HF-L group was significantly lower than that in the HF-S group.

During weight regain, when energy intake was not restricted and mice were refed a high-fat diet for one week, the expression levels of SCD1, SREBP1c, and its downstream target genes in the epididymal adipose tissue of mice, except ME, were significantly increased in the HF-L group (Figure 4C). Interestingly, when sea cucumber saponin supplements were no longer provided, the HF-S group had no significant difference except that the expression of FAS was 1.3 times higher than that of the HF group. The results showed that resumption of a high-fat diet after a calorie-restricted diet led to increased expression of lipid synthesis genes in adipocytes, resulting in increased body fat. It has been reported that SREBP-1c and FAS mRNA expression in white adipose tissue were tremendously increased in caloric restriction/refed rats, which was in line with our study [26,27]. When dietary supplementation of sea cucumber saponins was stopped, lipid synthesis in white adipose tissue was not up-regulated. In contrast, SREBP-1c-mediated lipid synthesis was significantly increased in white adipose tissue after calorie restriction and restoration of a full energy diet. This may be related to the fact that obese people try to lose weight by temporarily limiting their energy intake, but their weight and body fat rate recover quickly after they return to a normal diet. If the weight regain time was extended, the white adipose mass and body weight of the mice in the energy restriction group might have a higher rebound rate.

### 2.4. Effects on the Serum and Liver Lipid

**Figure 5:**
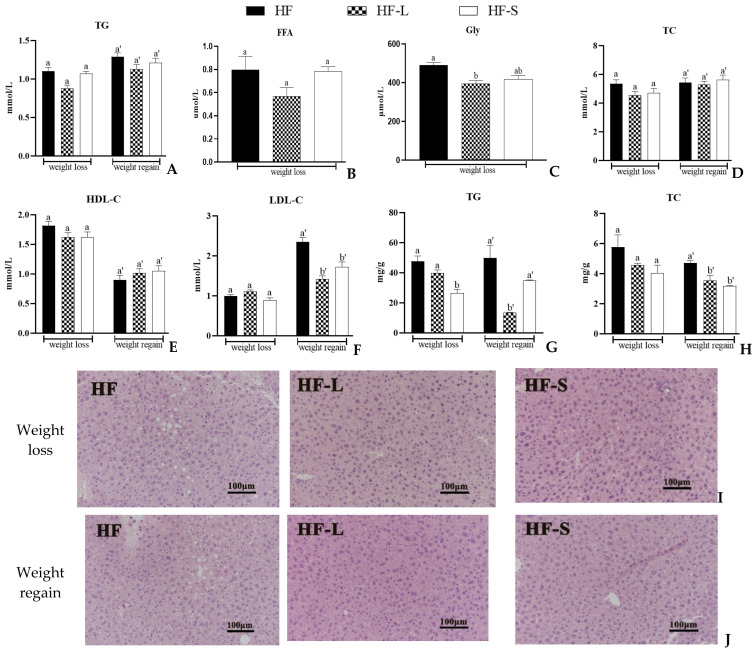
Serum contents of TG (**A**), FFA (**B**), Gly (**C**), TC (**D**), HDL-C (**E**), and LDL-C (**F**) as well as the hepatic contents of TG (**G**) and TC (**H**). Morphological changes in the liver by H&E staining during weight loss (**I**) and weight regain (**J**). Data reflect the mean ± SEM. Different letters a, b during weight loss and a’, b’ during weight regain indicate significant differences at *p* < 0.05. HF, high-fat group; HF-L, limited high-fat diet; HF-S, high-fat diet with saponins.

Figure 5A–H shows the effects of dietary sea cucumber saponin supplementation and energy restriction on serum and liver lipids, including serum TG, FFA, glycerin, TC, HDL-C, and LDL-C, and liver TG and TC. Interestingly, one week after resumption of the high-fat diet, compared with the weight loss period, serum TG increased by 28.4% in the HF-L group, 13.1% in the HF-S group, and 17.2% in the HF group (Figure 5A), which indicates that a two-week intervention of sea cucumber saponins exhibited a certain maintenance effect, even if sea cucumber saponins were no longer fed. During the weight loss, the low FFA in the HF-L group might be due to low food intake, but there was no significant difference among the three groups (Figure 5B). Studies have shown that serum FFA, TG, and TC levels were significantly reduced after 50% calorie restriction [28]. The glycerol level of HF-L was significantly lower than that of the HF group during weight loss, while there was no significant difference between the HF-S group and the other two groups (Figure 5C). There was no significant difference in serum TC and HDL-C levels during both weight loss and weight regain (Figure 5D,E). During weight regain, the serum LDL-C in the HF-S and HF-L groups was significantly lower than that in the HF group (Figure 5F). Interestingly, one week after the restoration of the high-fat diet, the TG of the liver of mice was significantly lower than that before the restoration (Figure 5G). There has been no previous study on this aspect, and the specific mechanism remains to be studied. The decrease in hepatic TC of the HF-S group during the weight regain might also be due to the maintenance effect of sea cucumber saponins (Figure 5H).

To further observe the results of hepatic fat accumulation, we analyzed the effects of energy restriction and sea cucumber saponins on lipid accumulation and hepatic steatosis by H&E staining (Figure 5I–J). During weight loss, there were no obvious lipid droplets in the liver of mice in the HF-L and HF-S groups, and a small number of lipid droplets could be seen in the HF group. In the weight regain period, compared with the weight loss period, a large number of lipid droplets could be seen in the liver of mice in the HF group. In the HF-L group, the liver lipid droplets almost disappeared after resuming the random high-fat diet. The number and size of liver lipid droplets in the HF-S group were still significantly less than those in the HF group, although they were slightly expanded. During weight regain, liver lipid accumulation in the HF-S group was slightly higher than that in the HF-L group, which corresponds to the liver TG (Figure 5G).

### 2.5. Effects on Fatty Acid Composition of Liver Lipids

The conversion of saturated fatty acids to monounsaturated fatty acids is the first step in lipid synthesis. The Δ^9^-desaturation index is the ratio of monounsaturated to saturated fatty acids (ratio of palmitoleic acid to palmitic acid (16:1/16:0) and oleic acid to stearic acid (18:1/18:0)) [29]. During weight loss, there was no significant difference in saturated fatty acids among the three groups (Table 2). Compared with the HF and HF-L groups, the 16:1/16:0 desaturation index of the HF-S group was decreased by 25% and 14.3%, respectively. Our previous studies showed that dietary sea cucumber saponins combined with exercise significantly reduced the liver desaturation index of mice [23]. Interestingly, despite the 40% restriction of energy intake, the desaturation index did not decrease significantly in the HF-L group, but instead increased slightly in 18:1/18:0 compared with the HF and HF-S groups. Possibly due to reduced energy intake, the mice were more inclined to store lipids rather than break them down. Studies have shown that after dietary calorie restriction of 60%, the rate of glucose oxidation is higher than that of fatty acid β-oxidation, and fatty acid β-oxidation as an energy supply method is delayed. The body relies more on glucose oxidation for energy supply, while fatty acids are stored [30]. During weight regain, the monounsaturated fatty acids C16:1 and C18:1 were significantly decreased in the HF-L group, and C18:0 was significantly increased. Compared with the HF group, the 16:1/16:0 and 18:1/18:0 desaturation indices of the HF-L group were reduced by 50% and 45%, respectively, and the HF-S group decreased by 12.5% and 25%, respectively. The 16:1/16:0 desaturation indices of the HF-L group were significantly lower than those of the HF-S group. The reason for the decreased desaturation index in the HF-L group after returning to high-fat diet may be that lipid storage is not required.

### 2.6. Effects on the Expression of Hepatic and Muscular Genes Involved in Lipid Metabolism

**Figure 6:**
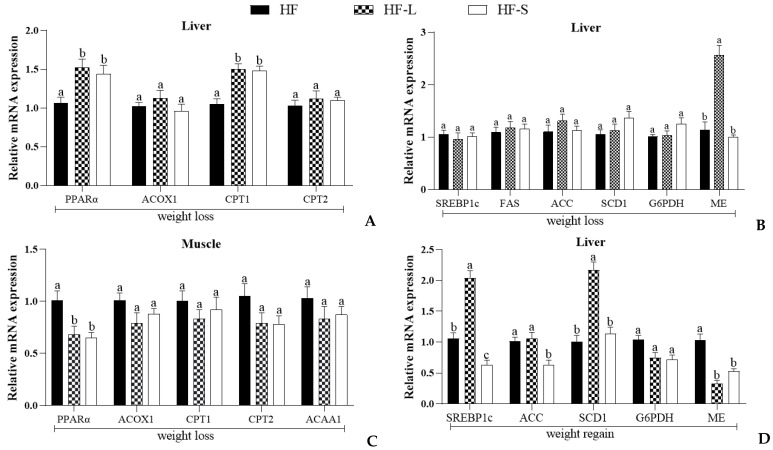
Effects of dieting and saponins on the mRNA expression of genes related to lipolysis, lipogenesis, and glucose metabolism in the liver and muscle of mice. The mRNA expression of PPARα, ACOX1, CPT1, and CPT2 in liver during weight loss (**A**). The mRNA expression of SREBP1c, FAS, ACC, SCD1, G6PDH, and ME in liver during weight loss (**B**). The mRNA expression of PPARα, ACOX1, CPT1, CPT2, and ACAA1 in muscle during weight loss (**C**). The mRNA expression of SREBP1c, ACC, SCD1, G6PDH, and ME in liver during weight regain (**D**). Data reflect the mean ± SEM. Different letters indicate significant differences at *p* < 0.05. HF, high-fat group; HF-L, limited high-fat diet; HF-S, high-fat diet with saponins.

After analyzing hepatic lipid and hepatic fatty acid composition, we analyzed the expression of genes related to lipid synthesis and lipolysis in the liver. Peroxisome proliferator-activated receptor (PPARα) is a highly ligand-activated nuclear receptor expressed in the liver, primordially identified as the molecular target of xenobiotic-inducing peroxisome proliferation in rodents [31]. Acyl-CoA oxidase 1 (ACOX1), a substrate of the first rate-limiting peroxisomal β-oxidation enzyme, is likely a PPARα agonist [32]. In rodents, long-chain fatty acid transport across the mitochondrial membrane is triggered by carnitine palmitoyltransferase-1 (CPT1) and carnitine palmitoyltransferase-2 (CPT2), whose proteins are localized in the outer and inner mitochondrial membrane, respectively [33]. The main function of CPT1 is to assist the transfer of long-chain fatty acids into the mitochondria through carnitine, and it is the rate-limiting enzyme for mitochondrial fatty acid oxidation. After that, fatty acids continue to be catalyzed by the CPT2 located in the inner mitochondrial membrane for oxidative decomposition. Figure 6A shows that energy restriction and the dietary addition of sea cucumber saponins both increased the mRNA expression of PPARα and CPT1 in liver during weight loss, and there was no significant difference between the HF-L and HF-S groups. It has been reported that the mRNA expression of PPARα and CPT1 in the liver of mice was slightly up-regulated after feeding a high-fat diet with 15% energy restriction for 6 weeks [34]. ME produces NADPH in the process of regulating malate metabolism, providing reducing power for fatty acid chain elongation. The expression level of ME in the liver of the HF-L group was significantly higher than that in the HF and HF-S groups during weight loss (Figure 6B). Therefore, it is speculated that lipid synthesis will be promoted during energy restriction, which is consistent with the previous experimental results of the liver fatty acid unsaturation index. Our previous study showed that sea cucumber saponins could inhibit the liver fatty acid synthesis caused by a high-fat diet, and the present result showed that saponins could promote PPARα-mediated liver fatty acid β-oxidation and exhibit no significant effect on liver lipid synthesis [23]. The difference in results may be due to the absence of a three-week fattening period, leading to lipid accumulation in the previous study. When liver lipid accumulation is excessive, dietary supplementation of saponins promotes liver lipolysis. We speculate that triglycerides transported to adipose tissue are reduced, which may be the reason for the decreased expression of adipose tissue lipolysis related mRNA (Figure 6A). Liver and muscle are the most active tissues for fatty acid oxidation, and the most important form of oxidation is β-oxidation [35]. Therefore, we determined the genes related to fatty acid β-oxidation in muscle (Figure 6C). The results showed that the mRNA expression of PPARα in the muscle of the HF-L and HF-S groups was significantly lower than that in the HF group during weight loss, and the downstream genes regulated by it were slightly decreased but not significantly different. Next, we determined gene expression for hepatic lipid synthesis during weight regain (Figure 6D). The results showed that the expression levels of SREBP1c and SCD1 in the HF-L group were significantly increased after resumption of the high-fat diet, while those in the HF-S group were still significantly lower than those in the HF group. The dietary supplementation of sea cucumber saponins may have a certain maintenance effect on the inhibitory effect on lipid synthesis.

### 2.7. Effects on the OGTT and Serum Parameters

**Figure 7:**
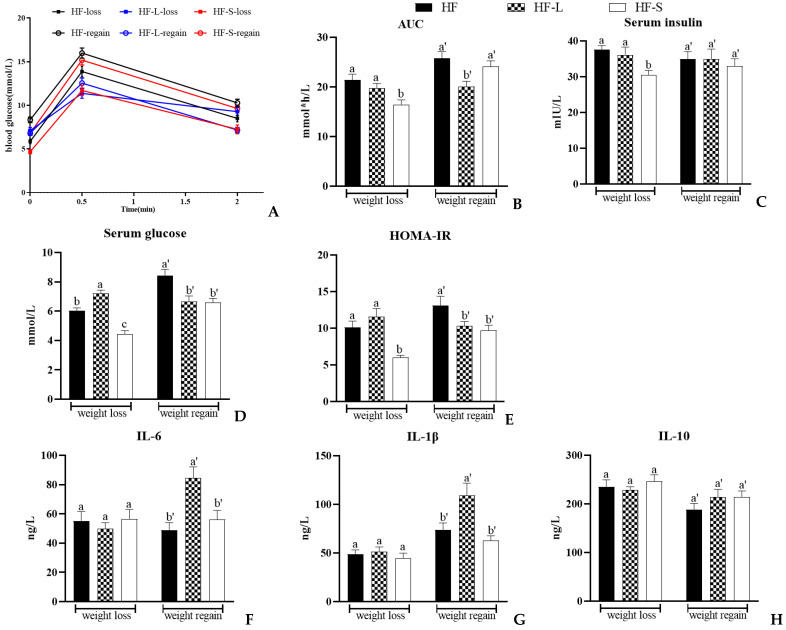
Effects of dieting and saponins on glucose tolerance (**A**), area under the curve (AUC) (**B**), serum insulin (**C**), glucose (**D**), HOMA-IR (**E**), IL-6 (**F**), IL-1β (**G**), and IL-10 (**H**) in mice. Data reflect the mean ± SEM. Different letters a, b, c during weight loss and a’, b’during weight regain indicate significant differences at *p* < 0.05. HF, high-fat group; HF-L, limited high-fat diet; HF-S, high-fat diet with saponins.

Growing evidence suggests that obesity activates inflammation by recruiting immune cells, such as macrophages and T cells, into tissues, leading to the development of insulin resistance [36]. Chronic low-grade inflammation leading to insulin resistance has been observed to be associated with high fat mass [37]. It is well known that adipocyte-derived interleukins (ILs) are involved in the development and progression of insulin resistance [38]. Figure 7 shows the effects of energy restriction and dietary sea cucumber saponins on the blood glucose, serum insulin, and insulin resistance in the mice in this study. When blood glucose levels among the three groups already differed in the fasting state, the area under the curve (AUC) for baseline glucose was calculated to validate the results (Figure 7A–E). During weight loss, the OGTT data revealed that glucose intolerance and insulin resistance were ameliorated by dietary sea cucumber saponins. Compared with the HF group and the HF-L group, the AUC of the HF-S group significantly decreased by 23.2% and 16.5%, and serum insulin decreased by 18.8% and 15.3%, respectively. Our previous study showed that dietary supplementation with 0.03% and 0.1% sea cucumber saponins alleviated the impaired glucose tolerance induced by a high-fat diet [39]. The fasting blood glucose of mice in the HF-S group was significantly lower than that in the HF and HF-L groups, and the HF-L group had the highest fasting serum glucose during weight loss. It has been reported that after 29 weeks of calorie restriction, fasting serum glucose was significantly lower in mice compared with the high-fat group [40]. The reason for inconsistency with this study may be the difference in experimental animals (C57BL/6 or KM) and the different energy limitation times. HOMA-IR value showed that the development of insulin resistance in the HF-S group was significantly lower than that in the HF and HF-L groups. During weight loss, sea cucumber saponins rather than energy restriction could reduce the serum insulin level and prevent the occurrence of insulin resistance, although the energy restriction could reduce the amount of white fat. During weight regain, both calorie restriction and sea cucumber saponins could reduce fasting serum glucose and significantly improve insulin resistance compared with the HF group.

In previous animal studies, under the condition of ensuring nutrition, reducing calorie intake by 40% improved the lifespan of animals, but in the state of “underfed”, the immune function of animals declined, and the risk of infection increased. Moreover, “starvation” will also make animals feel more stressed, and the secretion of cortisol in the body will increase, further suppressing immune function [41]. Figure 7F–H shows the contents of three cytokines involved in inflammatory reaction in the serum of the three groups. Clearly, during weight loss, there was no significant difference in IL-6, IL-1β, and IL-10 content among the three groups. However, after resuming the high-fat diet, IL-6 and IL-1β in the HF-L group were significantly higher than those in the HF and HF-S groups, while there was no significant difference between the HF-S and HF groups. It has been reported that weight regain after energy restriction correlated positively with inflammatory markers (IL-6) [42]. The results showed that returning to a high-fat diet after a period of energy restriction led to inflammation, whereas sea cucumber saponins did not.

## 3. Materials and Methods

### 3.1. Preparation and Analysis of Sea Cucumber Saponins

Saponins were extracted from sea cucumber (*Pearsonothria graeffei*), according to the previous methods of our laboratory [10]. Briefly, the body wall of the sea cucumber was crushed after vacuum freeze-drying and extracted with 60% ethanol (*v*/*v*), 4 times at room temperature. The ratio of material to liquid was 1:4. The combined extracts were evaporated in a vacuum and further separated using water and chloroform (1:1, *v*/*v*) to remove the liposoluble constituents. The aqueous layer was extracted with n-butanol, and the organic layer was vacuum evaporated to obtain n-butanol extract. The obtained n-butanol extract was dissolved in water and then placed on an HP-20 macroporous resin column using water and 80% ethanol as eluents. The obtained 80% ethanol extract was placed on a normal phase silica gel column and eluted with chloroform:methanol:water (10:1:0.1–7:3:0.3, *v*/*v*/*v*). Finally, the eluent (7:2.5:0.2, *v*/*v*/*v*) was mixed and concentrated in a vacuum to obtain sea cucumber saponin samples.

The obtained sea cucumber saponins were analyzed by high performance liquid chromatography (HPLC). HPLC-UV (Agilent 1260 Infinity system) analysis with an Eclipse XDB-C18 column (150 mm × 4.6 mm, 5 μm; Agilent, Palo Alto, CA, USA) was employed to evaluate the contents of the sea cucumber saponins at 205 nm. The temperature of the chromatographic column was kept at 30 °C. The chromatographic mobile phase consisted of acetonitrile (A) and 0.1% ammonium bicarbonate (B). Gradient elution was performed as follows: A: 0~5 min, 30%; 5~30 min, 30~60%; 30~35 min, 60~30%. The flow rate of the mobile phase was maintained at 1 mL min^−1^, and the injection volume was 20 μL. The purity of the obtained sea cucumber saponin sample was approximately 90%, including 23.8% HA and 65.5% EA (Figure S2).

### 3.2. Animal and Experimental Design

**Figure 8:**
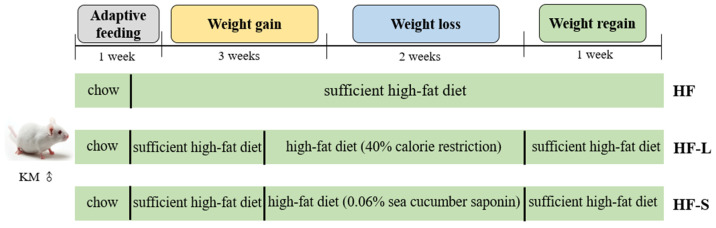
Experimental design and schedule of animal tests. n=8 male mice.

Eight-week-old male KM mice (18~20 g) were purchased from Vital River Laboratory Animal Technology Co Ltd. (Beijing, China). The mice were kept one per cage in a 12 h light/dark cycle at an ambient temperature of 22 ± 1 °C and a relative humidity of 60% ± 10%. The mice were acclimatized for 1 week and then provided with a high-fat diet for 3 weeks. They were then divided into three groups (n = 16) according to body weight, namely HF (high-fat diet group), HF-L (limited high-fat diet group), and HF-S (sea cucumber saponins group). Figure 8 shows the experimental design and schedule of animal tests. The three groups of mice all experienced a 2-week weight loss period. During the weight loss period, all three groups were provided with a high-fat diet that was regulated on the basis of AIN-93G, and their compositions are shown in Table S1. In addition, the HF-S group was supplemented with 0.06% sea cucumber saponins, and the dietary energy of the HF-L group was limited to 60% compared with that of the HF group. The mice underwent an oral glucose tolerance test (OGTT) at 11 days after weight loss and 6 days after weight regain. After the 2-week weight loss period, half of the mice in each group were sacrificed after fasting for 10 h. The remaining 8 mice in each group were all provided with a high-fat diet during weight regain and sacrificed one week later. The liver, kidney, heart, spleen, epididymal adipose tissue, perirenal adipose tissue, mesenteric adipose tissue, and subcutaneous adipose tissue were collected, weighed, frozen in liquid nitrogen, and stored at −80 ℃ for analysis. The animals were handled according to the guidelines provided by the Ethics Committee of Experimental Animal Care at the Ocean University of China (Qingdao, China, approval no. SPXY20190106, approved on 6 January 2019). The principles of care for laboratory animals were strictly adhered to, and all procedures were carried out in accordance with the guidelines formulated by the National Institutes of Health (NIH), and every effort was made to minimize the pain.

### 3.3. Oral Glucose Tolerance Test

Before the glucoses tolerance test, mice were fasted without water for 10 h. After oral administration of 0.2 g mL^−1^ glucose (2 g per kg body weight), blood was collected from the tail vein of mice at 0, 0.5, and 2 h, respectively. The blood was centrifuged for 15 min at 4 °C and 7500 r min^−1^. The serum glucose content was measured according to the instructions of the kit from Biosino (Beijing, China). The glucose tolerance curve was drawn, and the area under curve (AUC) was calculated.

### 3.4. Histological Analysis of Liver and Epididymal White Adiposes

Liver and epididymal white adiposes were harvested and fixed in 10% formalin, embedded in paraffin, and then dehydrated with graded alcohol. Tissue sections (5 μM thick) were stained with hematoxylin-eosin (H&E) and viewed by a light microscope equipped with a camera (Ni-E, Nikon, Japan).

### 3.5. Biochemical Analysis of Serum

Blood was collected and centrifuged at 4 °C for 3500 r min^−1^ for 15 min to obtain serum. Serum triacylglycerol (TG), cholesterol (TC), high-density lipoprotein cholesterol (HDL-C), and low-density lipoprotein cholesterol (LDL-C) concentrations were determined using enzymatic reagent kits from Biosino (Beijing, China). Serum-free fatty acid (FFA) and glycerol concentrations were determined using enzymatic reagent kits from Nanjing Jiancheng (Nanjing, China). Serum insulin was determined by enzyme-linked immunosorbent assay (ELISA, Invitrogen, Carlsbad, CA, USA). Serum glucose content was measured by via a kit from Biosino (Beijing, China). The homeostasis model assessment of insulin resistance (HOMA-IR) was calculated from insulin and glucose values according to the following formula: HOMA-IR = fasting blood glucose (mmol L^−1^) × fasting insulin (mU L^−1^)/22.5. Levels of serum interleukin (IL)-6, IL-1β, and IL-10 were determined by enzyme-linked immunosorbent assay (ELISA, Invitrogen).

### 3.6. Hepatic Lipid and Fatty Acid Composition Analysis

According to the method of Folch, hepatic lipids were extracted with chloroform-methanol 2:1, and the lower phase was the total pure lipid extract [43]. The obtained hepatic lipid was then dissolved with Triton X-100. The concentrations of TG and TC in the liver were measured by enzymatic reagent kits (Biosino, China). The experiment was strictly operated according to the instructions of the kit. Hepatic fatty acid composition, following transmethylation with HCL/methanol (1:5, *v/v*), was analyzed using a gas chromatograph (Agilent 6890) coupled to a flame-ionization detector and an HP-INNOWAX capillary column (30 m × 0.32 mm × 0.25 μm). Nitrogen was used as a vector gas (1.2 mL min^−1^). The detector temperature was kept at 250 °C, and the injector temperature was maintained at 240 °C. The column box was heated from 170 °C to 240 °C at a rate of 3 °C min^−1^, and it was then maintained at this temperature for 15 min [44]. Fatty acid methyl ester (FAME) determination was based on the retention times of each component using 15:0 methyl ester as the internal standard.

### 3.7. RNA Extraction and Real Time (RT)-PCR

Total RNA was extracted from liver or epididymal adipose tissue with Trizol reagent. The real-time qPCR analysis was conducted according to the previously published method [23]. A total of 2 μg RNA was amplified with random primers and was then reversely transcribed into cDNA. The target gene was amplified using SYBR Green I Master Mix (Riche, Mannheim, Germany) in an iCycler IQ5 system (Bio-Rad Laboratories, Hercules, CA, USA). The settings of the real-time PCR instrument were: 95 °C, 10 min, 1 cycle; 95 °C, 15 s, 45 cycles; 55–60 °C, 20 s; 72 °C, 30 s. The primers were synthesized by Shanghai Sangon biological Co., Ltd. The 2^−ΔΔCt^ method was used to calculate the relative change of gene expression, and GAPDH was used as the reference gene. The results were shown as relative changes with the HF group.

### 3.8. Statistical Analysis

All data were expressed as means ± standard error of the mean (SEM). The statistical differences between the groups were evaluated using 1-factor ANOVA with a least significant difference test by SPSS; *p* < 0.05 was considered statistically significant in all groups.

## 4. Conclusions

During the weight loss, adding 0.06% sea cucumber saponins to the diet and limiting energy intake by 40% can achieve a certain weight loss effect, and the effect is basically the same. However, energy restriction cannot avoid the insulin resistance caused by a high-fat diet, while dietary sea cucumber saponins can alleviate impaired glucose tolerance and insulin resistance. Interestingly, after resuming a high-fat diet, sea cucumber saponins still inhibit hepatic lipid synthesis through the SREBP1c pathway, even if saponins are no longer ingested. On the contrary, resuming the high-fat diet after limiting energy intake tends to synthesize lipids in the liver and white adipose tissue, and the immune system is threatened. Our obtained findings show that sea cucumber saponins can be used as a dietary supplement to reduce weight and alleviate impaired glucose tolerance and insulin resistance caused by obesity. Dietary sea cucumber saponins are expected to make up for the rapid weight regain and inflammation caused by calorie restriction, which is difficult for humans to adhere to. It should be noted that how long the effect of saponins on weight maintenance lasts after stopping dietary supplementation still needs further research.

## Figures and Tables

**Table 1:** Effects of dieting and saponins on body weight gain and visceral weight in mice.

Parameters	Weight Loss			Weight Regain		
	HF	HF-L	HF-S	HF	HF-L	HF-S
Food intake (g d^−1^)	4.60 ± 0.12 ^a^	2.90 ± 0.00 ^b^	4.55 ± 0.15 ^a^	4.45 ± 0.18 ^a’^	4.76 ± 0.22 ^a’^	4.43 ± 0.18 ^a’^
Initial body weight (g)	43.46 ± 0.94 ^a^	41.99 ± 0.83 ^a^	42.35 ± 0.84 ^a^	48.71 ± 2.18 ^a’^	38.33 ± 1.05 ^b’^	44.55 ± 0.84 ^a’^
Final body weight (g)	46.58 ± 1.23 ^a^	38.49 ± 0.73 ^b^	43.86 ± 0.78 ^a^	49.76 ± 2.32 ^a’^	45.80 ± 1.15 ^a’^	47.72 ± 1.30 ^a’^
Body weight gain (g)	3.14 ± 0.38 ^a^	−3.24 ± 0.29 ^c^	1.29 ± 0.52 ^b^	1.38 ± 0.43 ^c’^	8.37 ± 0.49 ^a’^	3.60 ± 0.72 ^b’^
Liver (g per 100 g BW)	3.34 ± 0.15 ^a^	3.48 ± 0.06 ^a^	3.32 ± 0.06 ^a^	3.46 ± 0.21 ^a’^	3.44 ± 0.03 ^a’^	3.45 ± 0.10 ^a’^
Heart (g per 100 g BW)	0.40 ± 0.02 ^a^	0.46 ± 0.01 ^a^	0.44 ± 0.02 ^a^	0.41 ± 0.02 ^a’^	0.44 ± 0.01 ^a’^	0.43 ± 0.02 ^a’^
Kidneys (g per 100 g BW)	1.36 ± 0.04 ^a^	1.37 ± 0.06 ^a^	1.38 ± 0.07 ^a^	1.49 ± 0.04 ^a’^	1.44 ± 0.04 ^a’^	1.47 ± 0.05 ^a’^
Spleen (g per 100 g BW)	0.21 ± 0.02 ^a^	0.18 ± 0.01 ^a^	0.20 ± 0.01 ^a^	0.25 ± 0.04 ^a’^	0.23 ± 0.02 ^a’^	0.27 ± 0.04 ^a’^

Values reflect the mean ± SEM of mice. Different letters a, b, c during weight loss and a’, b’, c’ during weight regain indicate significant differences at *p* < 0.05. HF, high-fat group; HF-L, limited high-fat diet; HF-S, high-fat diet with saponins.

**Table 2:** Fatty acid composition of hepatic lipids.

Fatty Acid Composition%	Weight Loss			Weight Regain		
	HF	HF-L	HF-S	HF	HF-L	HF-S
C16:0	23.21 ± 1.8 ^a^	23.41 ± 3.28 ^a^	24.02 ± 2.64 ^a^	22.92 ± 2.52 ^a’^	22.95 ± 2.98 ^a’^	24.48 ± 2.69 ^a’^
C16:1	1.76 ± 0.11 ^a^	1.56 ± 0.06 ^a^	1.46 ± 0.06 ^a^	1.81 ± 0.13 ^a’^	0.87 ± 0.08 ^b’^	1.60 ± 0.10 ^ab’^
C18:0	13.42 ± 1.48 ^a^	12.62 ± 1.51 ^a^	13.53 ± 1.62 ^a^	11.26 ± 1.35 ^b’^	16.02 ± 1.28 ^a’^	13.15 ± 1.58 ^ab’^
C18:1	19.85 ± 0.79 ^a^	21.05 ± 1.89 ^a^	18.51 ± 2.22 ^a^	22.55 ± 2.93 ^a’^	17.59 ± 1.23 ^b’^	19.67 ± 2.36 ^ab’^
C18:2	19.14 ± 2.10 ^a^	20.51 ± 1.64 ^a^	18.61 ± 1.30 ^a^	21.37 ± 1.92 ^a’^	17.62 ± 2.64 ^b’^	18.68 ± 1.49 ^ab’^
C18:3	0.60 ± 0.02 ^a^	0.61 ± 0.04 ^a^	0.52 ± 0.02 ^a^	0.90 ± 0.05 ^a’^	0.77 ± 0.05 ^a’^	0.86 ± 0.11 ^a’^
C20:4	14.80 ± 1.33 ^a^	13.70 ± 1.78 ^a^	15.76 ± 1.42 ^a^	12.78 ± 1.79 ^b’^	17.05 ± 1.88 ^a’^	14.45 ± 1.73 ^ab’^
C22:2	1.67 ± 0.05 ^a^	1.13 ± 0.00 ^b^	1.46 ± 0.07 ^a^	1.32 ± 0.11 ^a’^	1.29 ± 0.17 ^a’^	1.19 ± 0.10 ^a’^
C22:6	5.55 ± 0.39 ^a^	5.41 ± 0.32 ^a^	6.13 ± 0.31 ^a^	5.10 ± 0.71 ^a’^	5.84 ± 0.82 ^a’^	5.92 ± 0.71 ^a’^
C16:1/C16:0	0.08 ± 0.00 ^a^	0.07 ± 0.00 ^a^	0.06 ± 0.00 ^b^	0.08 ± 0.00 ^a’^	0.04 ± 0.00 ^b’^	0.07 ± 0.01 ^a’^
C18:1/C18:0	1.48 ± 0.13 ^a^	1.67 ± 0.22 ^a^	1.37 ± 0.16 ^a^	2.00 ± 0.14 ^a’^	1.10 ± 0.09 ^b’^	1.50 ± 0.15 ^b’^

Values reflect the mean ± SEM of mice. Different letters a, b during weight loss and a’, b’ during weight regain indicate significant differences at *p* < 0.05. HF, high-fat group; HF-L, limited high-fat diet; HF-S, high-fat diet with saponins.

## Data Availability

Not applicable.

## Supplementary Materials

The following supporting information can be downloaded at: https://www.mdpi.com/article/10.3390/md20100629/s1, Figure S1: Changes in the body weight of KM mice during weight loss (A) and weight regain (B); Figure S2: Analysis of Holothurin A (HA) and Echinoside A (EA) by HPLC; Table S1: Composition of experimental diet.
