# Supplementary material for: A Comparative Study of the Anti-Obesity Effects of Dietary Sea Cucumber Saponins and Energy Restriction in Response to Weight Loss and Weight Regain in Mice
Source: Mar Drugs. 2022 Oct 1;20(10):629. doi: 10.3390/md20100629 (PMC9605201; doi:10.3390/md20100629)
Supplement: Supplementary file 1 [file marinedrugs-20-00629-s001.zip › marinedrugs-1895126-supplementary.pdf]

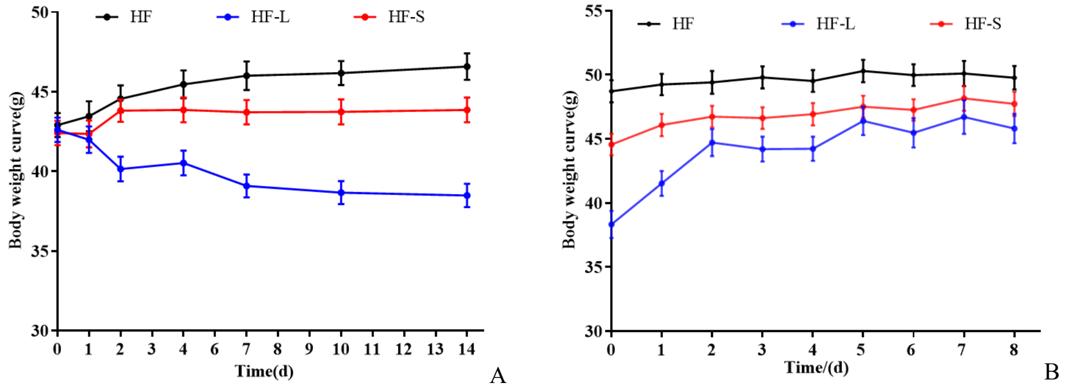

Figure S1. Changes in the body weight of KM mice during weight loss(A) and weight regain(B). Data reflect the mean ± SEM. Different letters indicate significant differences at P < 0.05. HF, high-fat group; HF-L, limited high-fat diet; HF-S, high-fat diet with saponins.

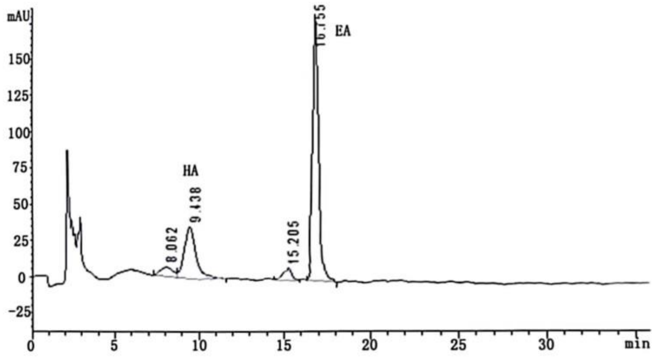

Figure S2. Analysis of Holothurin A (HA) and Echinoside A (EA) by HPLC.

Table S1. Composition of experimental diet (g/kg diet)

Content（g/kg）	HF	HF-L	HF-S
Casein	200	200	200
Corn starch	250	250	250
Saccharose	200	200	200
Corn oil	50	50	50
Lard oil	200	200	200
Mineral substance	35	35	35
Vitamin	10	10	10
Cellulose	50	50	50
Choline tartrate	3	3	3
DL-Methionine	2	2	2
Saponins	--	--	0.6

Note: "--", none add.
